# Surfactant-mediated Biodegradation of Polycyclic Aromatic Hydrocarbons

**DOI:** 10.3390/ma2010076

**Published:** 2009-02-23

**Authors:** Jing-Liang Li, Bing-Hung Chen

**Affiliations:** 1 Centre for Micro-Photonics, Faculty of Engineering and Industrial Sciences, Swinburne University of Technology, Hawthorn, Victoria 3122, Australia; 2 Department of Chemical Engineering, National Cheng Kung University, 1 University Road, Tainan 70101, Taiwan

**Keywords:** Surfactant, solubilization, polycyclic aromatic hydrocarbons, biodegradation and bioremediation

## Abstract

Polycyclic aromatic hydrocarbons (PAHs) are toxic environmental pollutants that are known or suspected carcinogens or mutagens. Bioremediation has been used as a general way to eliminate them from the contaminated sites or aquifers, but their biodegradation is rather limited due to their low bioavailability because of their sparingly soluble nature. Surfactant-mediated biodegradation is a promising alternative. The presence of surfactants can increase the solubility of PAHs and hence potentially increase their bioavailability. However, inconclusive results have been reported on the effects of surfactant on the biodegradation of PAHs. In this work, surfactant-mediated biodegradation of PAHs is reviewed.

## Prologue

Polycyclic aromatic hydrocarbons (PAHs) are hydrocarbons with fused benzene rings. The molecular structures of some typical PAHs are given in Table 1. The aqueous solubility of PAH decreases approximately one order of magnitude for each additional ring. For example, the aqueous solubility of naphthalene is about 30 mg/L and that of phenanthrene is only about 1 mg/L, while it is reduced to 0.1 mg/L for pyrene. The low aqueous solubility of PAHs limits their bioavailability and thus the efficiency of a bioremediation process. Motivated by the dramatic solubilization capacity of surfactants for hydrophobic compounds, surfactant-mediated bioremediation has been a research focus in recent years [1,2,3,4,5,6,7]. It’s generally assumed that the micellar phase of surfactants serves as a source of substrate. As the microorganism gradually depletes the hydrocarbons in the aqueous phase, the micelle-solubilized hydrocarbons diffuse into the aqueous phase. However, both positive and negative effects of surfactants on the degradation of hydrocarbons have been reported.

**Table 1:** The molecular structures of some typical PAHs.

Name of PAH	Molecular structure
Naphthalene	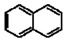
Phenanthrene	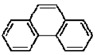
Anthracene	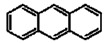
Pyrene	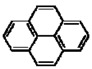
Benz[b]anthracene	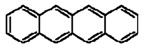
Triphenylene	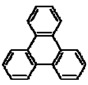
Pentacene	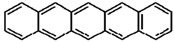
Benzo[a]pyrene	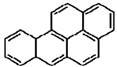
Coronene	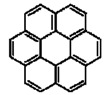

## 1. Solubilization by surfactant

**Figure 1:**
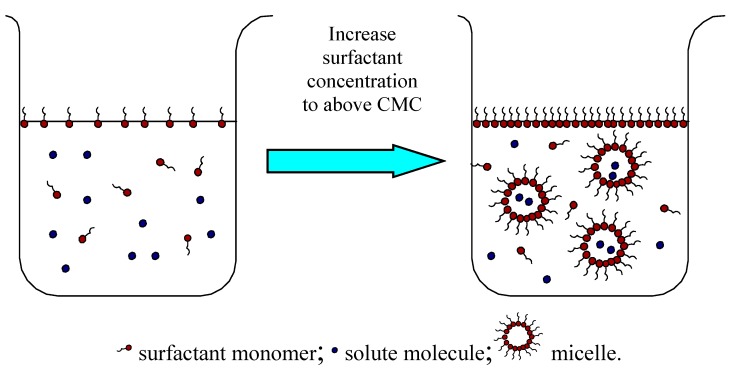
Micelle formation in water and solubilization. When the surfactant concentration is increased to above CMC, the surfactant monomers will associate with each other to form dynamic micelles. Depending on their polarity, the solute molecules will be solubilized into the core of the micelles or at the core-water interface.

In general, a surfactant molecule consists of a hydrophilic headgroup and one or two hydrophobic parts. The hydrophobic tail, usually a long hydrocarbon or fluorocarbon chain, acts to reduce the solubility of surfactant in water while the polar head has the opposite effect. The unique amphiphilic structures and properties of surfactants contribute to their versatility in numerous applications. Surfactant molecules can accumulate along the air-liquid and liquid-liquid interfaces and thus reduce both surface tensions and interfacial tensions at the same time. In addition, if the surfactant concentration exceeds a certain threshold, called the critical micelle concentration (CMC), at temperature higher than its Krafft temperature, surfactant monomers in aqueous solution will aggregate to form micelles of colloidal-size. Under such a condition, the hydrophobic solubilizates are incorporated into the hydrophobic cores of the micelles, which is called solubilization. More explicitly, solubilization may be defined as the spontaneous dissolving of a substance by reversible interaction with the micelles of a surfactant in a solvent to form a thermodynamically stable isotropic solution with reduced thermodynamic activity of the solubilized material [8]. The solubilization process can be described in Figure 1.

At surfactant concentrations above the CMC, the solubility of hydrocarbon increases linearly with surfactant concentration. Quantitative studies on the solubilization capacities of surfactants and effects of various parameters on the solubilization capacity of surfactants could be found in the literatures [9,10,11]. Apart from the solubility, the dissolution kinetics of hydrocarbons is also an important factor determining their biodegradation. This becomes more important when it comes to the remediation of weathered soils. The effects of surfactants on the dissolution kinetics of hydrocarbons from various matrices have also been reported. Surfactants are able to improve the mass-transfer of hydrophobic pollutants from a solid or non-aqueous liquid phase into the aqueous phase by decreasing the interfacial tension and by accumulating the hydrophobic compounds in the micelles [12,13].

## 2. Delivery of solubilized substrates into cells

**Figure 2:**
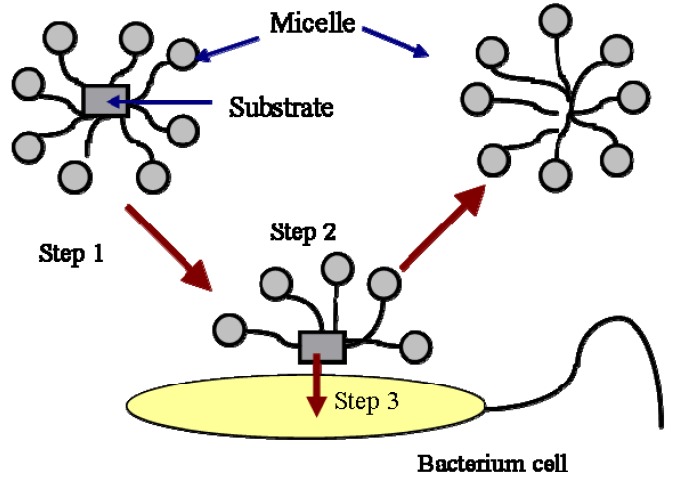
Uptake of substrate in micelles by a bacterial cell.

The biodegradation of hydrocarbons includes the degradation of hydrocarbon molecules in both the aqueous phase and the micellar phase. The biodegradation of hydrocarbon molecules is controlled by the diffusion of the molecules to the cell surface or enzyme sites. The molecules in the micellar phase are degraded either by first diffusing into the aqueous phase and then are utilized by the bacteria or by directing microbial uptake from the micelles. The first process is controlled by the kinetics of micellar aggregation. The relaxation time of the micelle is typically on the order of milliseconds to microseconds. Therefore, the first process is normally not a rate-limiting step. The second process is described in Figure 2.

As shown in this Figure, the mass transfer from micelle into cell is composed of three steps. The first step is the transport of the micelles solubilized with a substrate to the vicinity of the cells or enzymes by mixing. The second step is the exchange of the filled micelles with the hemimicellar layer of surfactant molecules formed around the cells. The formation of hemi-micelle layer around the cell or other substrates has been proposed and used successfully by many authors to describe the biodegradation [14,15,16] and dissolution of PAH [12,17]. The third step is the transfer of the substrate from the hemi-micelle to the cell. In a well-stirred system, the first step is also not a rate limiting step. The second and the third step normally control the biodegradation of a substrate in the micellar phase. The process is affected by the specific interactions between the micelle and the cell surface. It has been reported that the specific interaction between the micelle and the cell surface, such as the affinity of the two surfaces, is a factor controlling the transport of the substrate from the micelle to the cell [15,16,18,19].

## 3. Effects of surfactants on biodegradation of PAHs

Although it is agreed that surfactants can enhance the solubility and dissolution of hydrocarbons from contaminated soil [20,21,22,23,24], contradictory results have been reported on the ability of surfactants to enhance the biodegradation of hydrocarbons. The focus is whether solubilization is conducive or inhibitory to the microbial uptake of hydrocarbons. The enhanced biodegradation in the micellar solution can be attributable to the increased solubility and bioavailability of substrate to bacteria [5,12,25,26,27,28,29,30,31,32,33,34,35,36], surfactant-enhanced substrate transport through the microbial cell wall [37,38], increased interfacial area in the presence of surfactant [39], enhanced contact of bacteria with the hydrocarbon-water interface [40], facilitated direct contact between cells and non-aqueous liquid phase [41], and decreased diffusion path length between the site of adsorption and site of bio-uptake by the microorganism due to enhanced adsorption of cells to hydrocarbon occupied soil particles in the presence of surfactant [42]. Tiehm and Frizsche studied the biodegradation of both single and mixture of PAHs presolubilized by surfactant [43]. Accelerated biodegradation rates were found for both single and mixed PAHs presolubilized compared with the rate of PAHs in crystal form. This indicated that solubilization increased the bioavailability of PAHs.

The inhibitory effect was normally observed at surfactant concentrations approaching and exceeding the CMC. Potential mechanisms of inhibition include toxicity of surfactant to the microorganism [44,45,46], preferable microbial uptake of surfactants as substrate [47,48], and inhibition of the direct contact between cells and hydrocarbon by surfactant micelles [49,50]. It was also observed that the effect of surfactant was also dependent on the specific bacteria involved [18,19], which means that the specific interactions between bacteria and surfactant also play an important role. Some reported effects of surfactants on the biodegradation of PAHs are summarized in Table 2. It shows that several factors contribute to the overall effects of surfactants. For a same culture, different surfactants may have different overall effects [18,51], and, likewise, for a same surfactant, the overall effect differs when different microorganisms are involved [19].

**Table 2:** Some reported effects of surfactant on biodegradation of polycyclic aromatic hydrocarbons (PAHs).

Ref.	PAH	Surfactants	Microorganism	Effects	Explanation
[27]	Naphthalene	Brij30, Triton X-100	RET-PA-101(mixed culture isolated from contaminated wastes and soils)	+	Surfactants enhanced solubilization
[54]	Naphthalene	Triton X-100, PLE10	*Pseudomonas strain* 8909N	+	Surfactants enhanced dissolution
[19]	Naphthalene and Phenanthrene	Triton X-100	*Pseudomonas strain* 9816/11, *Sphingomonas yanoikuyae* B8/36	+ -	Surfactant had different effects on activity of different bacteria
[51]	Phenanthrene	Triton X-100 SDS, Tween 80 Tween 20	*Pseudomonas sp*. ZJF08	+ + -	Tween 20 was negative due to its preferable degradation by bacteria
[55]	Phenanthrene	Tergitol NP-10	*Pseudomonas stutzeri* P16	+	Surfactant increased dissolution rate of phenanthrene
[56]	Phenanthrene	Alfonic 810-60 Novel II 1412-56	Phenanthrene-degrading enrichment culture	+ +	Surfactant enhanced adsorption of phenanthrene
[18]	Pyrene Fluoranthene Benzo[a]pyrene	Brij 35 Igepal CA-630 Triton X-100 Tergitol NP-10 Tyloxapol	*Stenotrophomonas maltophilia* VUN 10,010	+ - + + +	Igepal CA-630 inhibited bacterial growth
[57]	Phenanthrene	Tween 20, SDS, TTAB, Citrikleen	pseudomonas aeruginosa	-	Toxicity of surfactant and solubilized substrate to bacteria
[58]	Naphthalene and Phenanthrene	SDS, SDBS T-maz-80, CA-620	Microorganism acclimated to naphthalene	-, 0	Competitive degradation between surfactants and PAHs
[59]	Phenanthrene and Pyrene	Tween 80	Agropyron elongatum	+	Surfactant enhanced solubilization
[60]	Anthracene	Biosurfactant	Bacillus circulans	+	Enhanced solubilization
[61]	Naphthalene, Phenanthrene, Pyrene, Fluoranthene	Tween 80	Phenanthrene degrading consortium	+	Increased solubility
[3]	Fluoranthene	Tween 80 and JBR (biosurfactant)	*Pseudomonas alcaligenes* PA-10	+	Enhanced desorption of PAH from soil
[62]	Naphthalene and Phenanthrene	Brij 30, Tween 80 and Triton X-100	Cultures isolated from wastewater site	+	Enhanced solubilization of PAHs
[5, 63]	Phenanthrene	Tergitol 15-S-X (X=7,9 and 12)	*Neptunomonas naphthovorans*	+/-	High surfactant concentration was not beneficial to bacteria
[64]	Pyrene	Tween 80	*Penicillium sp.*	+	Surfactant enhanced desorption
[31]	Pyrene, Fluoranthene, and Phenanthrene	Tween 80	*Sphingomonas paucimobilis* EPA 505	+	Direct transport of PAHs from micelles to cells
[65]	Anthracene, phenanthrene, and naphthalene	Microemulsion formed by Triton X-100 and oils	*Escherichia coil* JM109 (pPS1778) recombinant strain	+	Enhanced solulization
[6]	phenanthrene	SDS mixed with Brij 35, Tween80, and Triton X-100	Bacteria isolated from activated sludge	+	Enhanced solubilization and biodegradation of phenanthrene
[33]	phenanthrene	Biosurfactant rhamnolipids	*Pseudomonas putida* CRE 7	+	Increased bioavailability by surfactant
[66]	phenanthrene	Brij 30 and Brij 35 Triton X100 and Triton N101	Mixed culture	-	Low bioavailability of substrate in micelles

Note: +: positive effects; -: negative effects; 0: no effect.

## 4. Mechanisms of surfactant effects on biodegradation of PAHs

The positive effects of surfactants on hydrocarbon biodegradation have been invariably attributed to the increased solubility and dissolution of hydrocarbons or enhanced mass transport in the presence of surfactants. In contrast, there are several factors that contribute to the negative effects of surfactants on the biodegradation of hydrocarbons.

### 4.1. Toxicity of surfactants

Surfactants that are toxic to bacteria will inhibit cell proliferation and thus reduce their efficiency in degrading PAHs. Toxic surfactant molecules can induce cell apoptosis or necrosis depending on the concentration of the surfactant [52,53]. The surfactant molecules at a high concentration (near or above CMC) may form mixed micelles with membrane lipids, which may solubilize cell membranes. This will lead to the necrosis and lysis of cells. At concentrations below the CMC, when the surfactant molecules can not form mixed micelles with lipid molecules, the incorporation of surfactant monomers into the cell membrane is enough to impair the barrier function of cell membrane. Under this instance, surfactant molecules interfere with the phospholipid bilayer on cell membrane, inducing some enzymatic disorders, or penetrate into the cell. Consequently, an apoptosis signal is triggered. The toxicity of a surfactant is also dependent on its molecular structure.

According to the ionizability of the polar head, surfactant can be classified into nonionic and ionic surfactants. Ionic surfactants could be further categorized into cationic, anionic and zwitterionic surfactant. Typical examples of surfactants are given below.

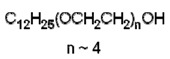
Polyethylene glycol dodecyl ether (Brij 30) (nonionic)
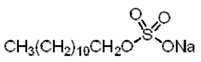
Sodium dodecyl sulfate (SDS) (anionic)
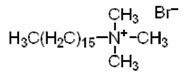
Hexadecyltrimethylammonium bromide (CTAB) (cationic)**C_12_H_25_N**^+^**(CH_3_)_2_CH_2_COO**^-^
*N*-Dodecylbetaine (zwitterionic)

Generally, nonionic surfactants are less toxic to microorganisms than ionic surfactants. The negatively charged surface of bacterial cells makes the cells more sensitive to the introduction of charged surfactants, especially positively charged cationic surfactants. The toxicity of a surfactant is also dependent on its molecular structure. It was observed by Tiehm that nonionic surfactants of the alkylethoxylate type and the alkylphenolethoxylate type with an average EO number of 9 to 12 monomers were toxic to several PAH-degrading cultures [13]. Toxicity decreased with increasing hydrophilicity (HLB) of the surfactants. The high water solubility of a surfactant with a higher HLB inhibits its entering into the lipid bilayer of cell membrane. A detailed study on the toxicity of surfactant to bacteria and on the biodegradation of phenanthrene was reported [67]. On the basis of experimental observation, the increasing order of toxicity of the studied surfactants is followed by non-ionic surfactants (Tween 80, Brij30, 10LE and Brij35) < anionic surfactants (LAS) < cationic surfactants (TDTMA). The bacterial growth increased slightly when phenanthrene and LAS (≤ 10 mg L^-1^) served the sole carbon and energy resource. However, the degradation of phenanthrene showed no obvious change at lower surfactant concentrations due to the competitive utilization of the surfactant as a non-toxic substrate. At higher surfactant concentrations, the degradation of phenanthrene was decreased presumably due to the reduced microorganism activity. In others investigations, a similar trend of surfactant toxicity was also observed, *i.e.* nonionic surfactants< anionic surfactants < cationic surfactants [68,69]. The previous studies indicate that nonionic surfactants are better choices for enhancing the biodegradation of PAHs, due to their low cytotoxicity. However, they have different degrees of toxicity, depending on their molecular structure. Polyoxyethylene octyl phenols (Triton X series) have shown to be highly toxic to cells, because they can solubilize the membrane lipid bilayer [70]. In fact, Triton X-100 is used as a general agent for cell lysis. Polyoxyethylene sorbitan surfactants (Tween series) have been shown to possess low toxicity [3,36,59,67,71]. For the same head group and similar molecular structure, the toxicity to the bacteria is affected by the chain length of the hydrophilic moiety. Generally, surfactant toxicity becomes lower as the chain length increases (i.e. an increasing hydrophilicity or HLB) [67]. This is due to the high aqueous solubility of a surfactant with a higher HLB and less intensive interaction between its molecules and hydrophobic cell membrane.

### 4.2. Biodegradation of surfactants

Surfactant biodegradability is one factor that determines its applicability for *in-situ* bioremediation applications. If the surfactant is highly degradable to the microorganism, it may become a competitive carbon source, which influences the degradation of the primary substrate. Inhibited degradation of PAHs due to the preferable degradation of surfactant was reported [13]. On the other hand, the biodegradation of surfactant can lead to the release of more PAHs from the micellar phase into the aqueous phase, which increases their bioavailability. However, surfactants that can be readily degraded will quickly lose their solubilization capacity and render them ineffective for solubilization purpose. Therefore, in practical applications, balance has to be found between the biodegradability of the surfactants and their influence on the biodegradation of the pollutants. Suitable surfactants have to be prescreened before an *in-situ* bioremediation process to be carried out. Parameters to be considered include the solubilization capacity of the surfactants for the desired contaminates, physical properties of the surfactants such as its stability (clouding point, etc), and a suitable degree of biodegradability.

### 4.3. Bioavailability of solubilized PAHs

At surfactant concentrations above the CMC, the dissolved substrate is sequestered into surfactant micelles, which reduce their bioavailability. Since the micellar phase contains most of the dissolved substrate, the bioavailability of solubilized hydrocarbons decides the overall biodegradation of the substrate. The bioavailability of hydrocarbons in the micellar phase has been studied both qualitatively and quantitatively.

Tiehm reported that an exponential growth pattern was observed in mixed cultures on phenanthrene and fluoranthrene solubilized by a nonionic surfactant, which indicated the high bioavailability of the solubilized hydrocarbons [13]. Liu, *et al*. quantified the bioavailability of micelle-solubilized naphthalene to naphthalene-degrading microorganisms. Two nonionic surfactants, Brij 30 and Triton X-100 were used [27]. Results showed that naphthalene solubilized by surfactant micelles was bioavailable and degradable by the mixed bacterium cultures.

Guha and Jaffé studied the biodegradation kinetics of phenanthrene and PAH mixtures partitioned into the micellar phase of nonionic surfactants [16]. The bioavailability of phenanthrene was depicted by an effective concentration of phenanthrene in the micellar solution available for biodegradation. The effective concentration, *C_e_*, can be conceptually assumed to include the molecular phenanthrene dissolved in the aqueous phase and a fraction (*f*) of the phenanthrene solubilized in the micellar phase that can be directly accessed and consumed by the bacteria, i.e.

(1) $$C_{e}=C_{a}+f\cdot C_{mic}$$

where *f* is the bioavailability coefficient, ranging from 0 to 1, dependent on surfactant concentration; *C_a_* (mg phenanthrene/L bulk solution) and *C_mic_* (mg phenanthrene/L bulk solution) are the phenanthrene concentration of the aqueous phase and micellar phase respectively. With *f* = 1, *C_e_* = *C* and *f* = 0, *C_e_* = *Ca*. *C* (=*C_mic_*+*C_a_*) is the bulk concentration of phenanthrene in a surfactant solutions.

Experiments and modeling on a few surfactants showed that the value of the bioavailablity coefficient *f* depends both on the surfactant molecular structure and surfactant concentration. According to their results, the bioavailability coefficient of Brij 35 is zero. It means that the microoganism used in the experiments could not attack the phenanthrene in the micelles, whereas, the *f* values of polyethylene (9.5) glycol nonylphenyl ether (Triton N101), Triton X-100, and Brij 30 were between 0 and 1. With an increase in surfactant concentrations, *f* approached zero, indicating that the phenanthrene molecules in the micelles cannot be taken by the cells directly. In other words, the substrate molecules have to be transferred from the micellar phase into the aqueous phase to be bioavailable to the bacteria. They also suggested that *f* is not only a function of the surfactants, but most likely also of the bacterial culture. The authors also investigated the mechanism of bioavailability of hydrophobic compounds partitioned into the micellar phase of nonionic surfactant [15]. They also studied the bioavailability of PAH mixtures partitioned into the micellar phase of a nonionicsurfactant [14].

Zhang *et al*. studied the effects of biosurfactants on the dissolution, bioavailability, and biodegradation of phenanthrene [33]. A mathematical model was used to describe the combined effects of solubilization and biodegradation, and the bioavailability of phenanthrene within surfactant micelles. The bioavailability of substrate within micelles was found to depend on the surfactant used. Experimental results indicated that the effect of a surfactant on biodegradation was a combination of the solubilizing power of the surfactant and the bioavailability of the substrate within the surfactant micelles.

In study of biodegradation kinetics of the surfactant-solubilized fluoranthene, Willumsen and Arvin observed that surfactant-solubilized fluoranthene could promote the rate of fluoranthene degradation, but, to a lesser extent than expected projected on the observed surfactant-enhanced fluoranthene solubilization [72]. They suggested that the bioavailability of micelle-solubilized fluoranthene might be one factor controlling mineralization in such system.

Li and colleagues studied the biodegradation of phenanthrene in the presence of linear alcohol ethoxylate nonionic surfactants [5,63]. The bioavailability of phenanthrene in the micellar solution of the surfactants was investigated based on the solubilization extent of phenanthrene.

At solubilization equilibrium,

(2) $$C_{mic}=S_{mic}K_{mw}C_{a}$$

where *S_mic_* (mg/L) is the surfactant concentration in micellar phase; and *K_mw_* (L/mg) is the micelle-water partition coefficient of phenanthrene. The partition coefficient *K_mw_* is defined as the ratio of the phenanthrene concentration in micellar phase, *C_m_* (mg phenanthrene/ mg micellized surfactant) to its aqueous concentration *C_a_*, *i. e.*

(3) $$K_{mw}=\frac{C_{m}}{C_{a}}=\frac{C_{mic}}{S_{mic}C_{a}}$$

If define α as the fraction of a substrate partitioned into the micellar phase as solubilization extent, *i. e*. *α* = *C_mic_*/*C*. Eq.(3) can then be rewritten as

(4) $$K_{mw}=\frac{C_{m}}{C_{a}}=\frac{C_{mic}}{S_{mic}C_{a}}=\frac{\alpha C}{S_{mic}(1-\alpha )C}=\frac{\alpha }{S_{mic}(1-\alpha )}$$

A rearrangement of Eq. (4) gives:

(5) $$\frac{1}{\alpha }=1+\frac{1}{K_{mw}S_{mic}}$$

Eq.(5) indicates that the solubilization extent, α, increases with the surfactant concentration, *S_mic_,* and is independent of the phenanthrene concentration, *C*. This is understandable since the distribution of phenanthrene between the two phases, i.e. the aqueous and micellar phases, is determined by the partition equilibrium and the amount of micelles in the micellar solution.

From Eqs. (2) and (4), it arrives:

(6) $$C_{a}=\frac{C}{1+S_{mic}K_{mw}}$$

Introducing Eq. (2) and (6) into (1), the effective concentration can be written as

(7) $$C_{e}=\left[1-\left(1-f\right)\cdot \alpha \right]\cdot C$$

At a constant initial phenanthrene concentration *C* and with an increase in the surfactant concentration, *α* increases and *f* decreases. It can be seen from Eq.(7) that the effective concentration or the bioavailability of phenanthrene in the micellar solution decreases with the increase of surfactant concentration. Eq.(7) also indicates that, at a constant surfactant concentration (i.e. constant *α* and *f*), the effective concentration or the bioavailability of phenanthrene in the micellar solution increase with the initial phenanthrene concentration, *C.* Hence, Eq.(7) implies that biodegradation of phenanthrene in the micellar solution will be affected inversely by the concentration of the surfactant, and be enhanced by the initial phenanthrene concentration dissolved in the micellar solution. This conclusion is in fact in agreement with the experimental results.

**Figure 3:**
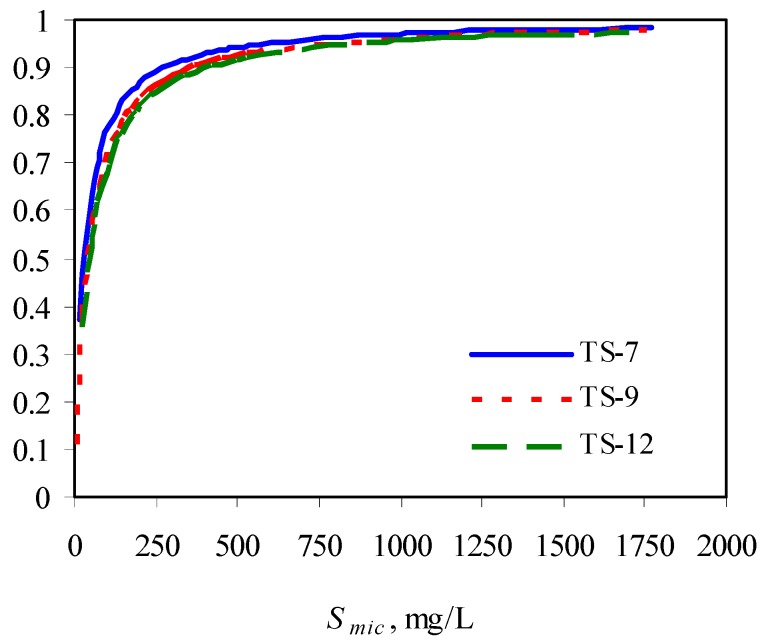
Solubilization extent as a function of micellized surfactant concentration. TS-X in the legend represents Tergitol 15-S-X.

The authors observed that the presence of surfactants enhanced the biodegradation of phenanthrene due to its increased solubility, compared with its biodegradation in the absence of surfactant. However, at a fixed initial phenanthrene concentration, with increase in surfactant concentration, its biodegradability was compromised, indicating the inhibitory effects of surfactant at higher concentrations. This could be due to the low availability of phenanthrene partitioned into the micellar phase. With increase in surfactant concentration, the solulization extent *α* increases sharply and then approach 1. This means at a high surfactant concentration, the substrate is mainly in the micellar phase. When the concentration of the micellized surfactant is above 300 mg/L, more than 90% of the total substrate is in the micelles (Figure 3) [73].

The lower bioavailability of the phenanthrene in the micelle contributed to its overall lower biodegradability at higher surfactant concentrations. This was indicated by an interesting observation of the authors. That is, the biodegradation of phenanthrene was enhanced after a period of plateau. The mechanism is that with loss of surfactant during the degradation process, more phenanthrene was released from the micellar phase into the aqueous phase, making it readily available to the bacteria.

The effects of surfactant on PAH biodegradation and vice versa were also studied by some other researchers [58]. It was observed that the biodegradation of naphthalene and phenanthrene were dependent on the surfactant used, and the presence of naphthalene and phenanthrene also influence the biodegradation of different surfactants to different degrees.

Garcia *et al*. studied the effect of the nonionic surfactant Brij 35 on the bioavailability of solid and Teflon-sorbed dibenzofuran for *Sphingomonas sp.* strain HH19K [74]. It was observed that the presence of this surfactant accelerated the dissolution and biodegradation of solid dibenzofuran by a factor of 2. However, it slowed down the initial biodegradation of desorbing dibenzofuran. They proposed that two processes might reduce the bioavailability of sorbed dibenzofuran. First, desorbing dibenzofuran rapidly accumulated in the surfactant micelles, which reduced dibenzofuran concentration in the aqueous phase, which controls the biodegradation rate. Second, Brij 35 suppressed the contact between bacteria and Teflon. This increased the average diffusion distance of dibenzofuran to the bacteria, which in turn flattened the gradient of the dissolved dibenzofuran concentration between the sorbent and the cells.

As aforementioned, the effect of a surfactant on the biodegradation of hydrocarbons is contributed by many factors simultaneously. The bioavailability of substrates in the micellar phase decides the overall performance of surfactants in the bioremediation process. Consequently, it is also the reason that much research work has been carried out in this aspect. For a successful field application, the selection of surfactants is probably the most important step. Such a surfactant should be nontoxic to the microorganism and pose no environmental concerns, also should have a good solubilization capacity for the targeted contaminants. All these factors together with the bioavailability of the compounds solubilized in the micelles of the surfactant should be examined before field applications. In addition, the surfactant selection must also consider operation factors, such as turbidity and foam generation [75].

### 4.4. Toxicity of PAHs at high concentrations due to solublization

It was observed that PAHs themselves could be toxic if presenting at high concentrations due to the solublization by surfactants [57,71]. Research by Bramwell and Laha showed that the presence of solubilized phenanthrene increased the toxicity of a nonionic surfactant Tween 20 by a 100-fold. This indicates that the toxicity of solubilized substrate also needs to be considered in the application of surfactant-mediated bioremediation [57]. In a recent work, the toxicity of solutions containing nonionic surfactants Tween 80, Brij 35 and/or phenanthrene to *Pseudomonas putida* ATCC 17484 was investigated. The fraction of phenanthrene in the surfactant solution that can be directly contacted by bacteria was evaluated. It was observed that this part of phenanthrene was toxic to bacterial cells. At a fixed surfactant concentration, the toxicity of the solution was increased by increasing phenanthrene concentration. The toxicity of a solution with a certain phenanthrene concentration could be reduced by increasing the surfactant concentrations to decrease the direct contact of bacteria with the phenanthrene solubilized in micelles. Similar results were reported by the authors using nonionic surfactants Tween 80 and Triton X-100, and another bacteria strain *Pseudomonas putida* P2 [76].

## 5. Ways to reduce the negative effects of surfactants

### 5.1. Biosurfactants as alternatives to synthetic surfactants

There is growing interest in the utilization of biosurfactants in enhancing the biodegradation of hydrocarbons [60,77,78,79,80]. Biosurfactants are biological compounds produced by microorganisms. Like synthetic surfactants, they exhibit high surface-active properties and their molecules can produce micelle or micelle-like aggregates. Typical biosurfactants include glycolipids, lipopeptides, fatty acids and polymeric compounds. The production of biosurfactants depends on a variety of factors such as the microorganism and nutrient sources. With the increasingly strict regulation on the use of environmentally compatible products, the use of biosurfactants in place of synthetic surfactants is increasing. Biosurfactants have a wide range of industrial applications in various fields including food, cosmetics, pharmaceutics, oil recovery and environmental remediation. Due to their good biocompatibility with the cell membrane, they are less toxic to microorganisms [81] than synthetic surfactants. Thus, it is a good alternative to synthetic surfactants. However, biosurfactants have not been utilized widely in industrial applications. The major disadvantage of using biosurfactants is the relative high production and recovery cost, as well as the difficulty of their mass production. To make the surfactant-mediated bioremediation cost-effective, efforts need to be put into the development of non-toxic synthetic surfactants mimicking the structure of the natural biosurfactants.

### 5.2. Enhancing surfactant tolerance of microorganisms

As discussed above, the toxicity of a surfactant is mainly due to its membrane susceptibility. Therefore, suitable additives can be searched to reduce the susceptibility of cells to surfactant. It was observed that the toxicity of Triton X-100 on *Sphingomonas paucimobilis* strain EPA505 can be significantly reduced in the presence of calcium ions (Ca^2+^) [82]. In the absence of Ca^2+^, Triton X-100 at a concentration of 0.48 mM (0.3g L^-1^) reduced cell viability by 100% and thus completely inhibited the biodegradation of fluoranthene. In the presence of 4.13 mM Ca^2+^, the cell viability was reduced only by 10% and the maximum mineralization rate of fluoranthene was more than doubled. It is likely that the Ca^2+^ ions can stabilize the cell membrane, making the cell less sensitive to the surfactant. Mg^2+^ was also observed to enhance the surfactant tolerance of the cell, but to a less extent than Ca^2+^.

### 5.3. Selection of suitable combinations of surfactant and microorganisms

As shown in Table 2, a certain strain of microorganism can respond differently to different surfactants which it is exposed to. This is due to the specific interactions between the surfactant molecules and cell membrane. Therefore, for a selected microorganism, prescreening work has to be done to make a choice of a suitable surfactant. Molecular simulation can also be adopted to predict the interaction between a surfactant and cell in surfactant selection. Nevertheless, other parameters of a surfactant especially its solublization capacity has to be taken into consideration.

## Summary

This work gives a review of surfactant effects on the biodegradation of solubilization of PAHs, a family of common and toxic pollutants that have raised significant environmental concerns. Surfactants have been proven to be important vehicles for the recovery of these compounds from contaminated soil or aquifers due to the solubilization process. Both positive and negative effects have been reported on surfactants on microbial utilization of PAHs. The positive effects are generally attributable to the increased solubility/dissolution these compounds by surfactants which enhances their bioavailability. The negative effects are contributed by a variety of factors, which include toxicity of surfactants to microorganism, preferential degradation of surfactants and limited bioavailability of substrate solubilized in surfactant micelles. Nonionic surfactants are normally less toxic to microorganisms than ionic surfactants due to the weaker interactions between the neutral surfactant molecules and charged cell membrane. For a bioremediation application, solubilization efficiency is a prior criterion for the selection of a surfactant. However, its biodegradability and toxicity to the microorganism have to be considered to ensure an efficient remediation and the environmentally friendly application of the surfactant. Other important parameters of surfactant to be considered include its soil adsorption and cloud point. Surfactants with moderate biodegradability to the microorganisms should be considered. Firstly, sufficient solubilization capacity of such a type of surfactant can be maintained during a bioremediation process. Secondly, the reduction of effective surfactant concentration can increase the bioavailability of the substrate by releasing them into the aqueous phase. Thirdly, surfactants with a certain degree of biodegradation are more environmentally benign. Selection of surfactants that are nontoxic or with minimal toxicity to microorganisms is also essential to achieve a successful bioremediation. Biosurfactants are good alternative to synthetic commercial surfactants in term of low cytotoxicity. However, their application is limited due to their small scale production. To make the surfactant-mediated bioremediation a cost effective technique, efforts should be taken on the development of synthetic surfactants that biologically compatible with cells.
